# Changes in transcript levels of starch hydrolysis genes and raising citric acid production via carbon ion irradiation mutagenesis of *Aspergillus niger*

**DOI:** 10.1371/journal.pone.0180120

**Published:** 2017-06-26

**Authors:** Wei Hu, Wenjian Li, Hao Chen, Jing Liu, Shuyang Wang, Jihong Chen

**Affiliations:** 1 Institute of Modern Physics, Chinese Academy of Sciences, Lanzhou city, Gansu Province, China; 2 College of food science and engineering, Gansu Agricultural University, Lanzhou city, Gansu Province, China; National Renewable Energy Laboratory, UNITED STATES

## Abstract

The filamentous ascomycete *Aspergillus niger* is well known for its ability to accumulate citric acid for the hydrolysis of starchy materials. To improve citric acid productivity, heavy ion beam mutagenesis was utilized to produce mutant *A*.*niger* strains with enhanced production of citric acid in this work. It was demonstrated that a mutant HW2 with high concentration of citric acid was isolated after carbon ion irradiation with the energy of 80Mev/μ, which was obvious increase higher than the original strain from liquefied corn starch as a feedstock. More importantly, with the evidence from the expression profiles of key genes and enzyme activity involved in the starch hydrolysis process between original strain and various phenotype mutants, our results confirmed that different transcript levels of key genes involving in starch hydrolysis process between original strain and mutants could be a significant contributor to different citric acid concentration in *A*.*niger*, such as, *amyR* and *glaA*, which therefore opened a new avenue for constructing genetically engineered *A*.*niger* mutants for high-yield citric acid accumulation in the future. As such, this work demonstrated that heavy ion beam mutagenesis presented an efficient alternative strategy to be developed to generate various phenotype microbe species mutants for functional genes research.

## Introduction

The efficient production of citric acid has drawn the attention of researchers because of its market demand and extensive applications as additive supplements in the food, cosmetic, and pharmaceutical industries [1, 2]. Citric acid can be produced via artificial extraction, chemical synthesis or microbial fermentation [3]. Currently, the development of microbial fermentation for producing citric acid has attracted significant attention due to its high citric acid productivity, and microbial fermentation accounts for approximately 90% of world's citric acid production [4, 5]. Previous studies have revealed that several microorganisms can accumulate citric acid, such as *Aspergillus niger*, *Penicillium janthinelun*, *Yarrowia lipolytica*, *Bacillus licheniformis* [6]. Of these, the filamentous fungus *A*.*niger* has attracted considerable interest. Critically, it has a unique ability to produce citric acid with high productivity from inexpensive starchy materials including yam starch [7], and corn meal hydrolysate [8, 9].

In order to improve citric acid productivity in *A*.*niger*’s fermentation from starchy materials, In addition to improvement in fermentation processes [6, 10], mutation breeding of citric acid-overproducing strains via random mutagenesis has been suggested as a valuable option. Conventional methods of mutagenesis including ultraviolet irradiation (UV), ethyl methanesulfonate (EMS) and acridine orange (AO), have been widely applied to breed citric acid-overproducing strains. In the study by Sarangbin and Watanapokasin [7], UV-induced mutant YW-112 achieved about 106 g/l at 5 days of cultivation time. In the study by Lesniak [11], *A*.*niger* B-64-5 after UV irradiation displayed citric acid production of 162 g/l after 188 h of shake-flask fermentation. Nevertheless, the citric productivity achieved by those mutant strains from starchy materials was still not enough to meet industrial demand.

Given the inherent characteristics and technological advantages, heavy ion beam mutagenesis has been employed as novel and efficient mutagenesis technology for breeding for plants or microbes than other mutagenic techniques such as chemical mutagens (EMS) and ionizing radiation (X-rays or -rays). Because heavy ion beam mutagenesis possesses high linear energy transfer (LET) and relative biological effectiveness (RBE) [12, 13], which can induce complex DNA damage, such as stable knockout (gene deletion) mutation, rearrangements, and translocations, which can establish abundant phenotype mutant libraries and offer a valuable strategy for studying functional gene [14, 15]. The breeding applications of high-LET heavy ion mutagenesis in fungi has been carried out, such as *A*.*niger* [16], *Aspergillus terreus* [17], *Trichoderma viride* [18], *Euglena gracilic* [19]. In our previous work, a promising *A*.*niger* mutant H4002 induced by heavy ion beam mutagenesis, has been reported, which can produce 187.5 g/L citric acid from corn starch hydrolyzate with extremely high productivity of 3.13 g/(L•h) [20]. This was also the highest reported citric acid production in bioreactor so far, showing significant value in industrial application [21]. Inspired by our previous work described as above, mutant strain H4002 was further induced by heavy ion irradiation mutagenesis in order to obtain different phenotypic *A*.*niger* mutants with variable citric acid production capacity in this work. This strategy can further improve the production yield of citric acid in *A*.*niger* from starchy materials. More importantly, several phenotypic *A*.*niger* mutants with variable citric acid production capacity could be valuable for investigations of the genetic mechanism of different citric acid accumulation in *A*.*niger*, which can provide a guideline for rational constructing genetically engineered *A*.*niger* mutants for high-yield citric acid accumulation in the future.

## Materials and methods

### 1: Strains and media

The original strain used in this work, named H4002, was obtained from carbon ion irradiation and was provided in IMP, CAS [20]. Mutants HW2 and H4 were obtained by carbon ion irradiations of *A*.*niger* H4002. The different carbon sources medium for citric acid accumulation was composed of 100 g/L corn starch or glucose, 12 g/L soybean meal, and pH = 6.0 H_2_O. The medium were composed of 150 g/L soluble starch and 12 g/L soybean meal for RT-PCR analysis and glucoamylase activity detection. All the media were autoclaved at 118°C for 30min.

### 2: The colony appearance of A.niger

Conidia of the mutants and the original strain cultivated on PDA-containing slopes for 6 d were streaked on the solid plate medium. The solid plate medium had the following composition: 200 g/L potato juice, 20 g/L sucrose, 20 g/L agar. The cultivation conditions were 36°C for 48 h and 72 h.

### 3: Real-time RT-PCR analysis

0.5mL conidial suspensions of both original strain and mutants (1.2×10^6^ conidia /mL) were added into fermentation media for 24h and 48h, respectively. Relevant mycelia of *A*.*niger* were collected, then flash-frozen in liquid nitrogen and ground into fine power. Total RNAs of both original strain and mutants were extracted by Trizol reagent (TaKaRa, Japan) according to the manufacturer's instructions, and the quality of RNAs were assessed using Smart spec^™^plus (BIO-RAD, USA). The obtained of DNase-I-treated RNA was used as a template to synthesize the *glaA* and *amyR* genes cDNA using Transcriptor First Strand cDNA Synthesis Kit. Real-time RT-PCR analysis was performed to determine transcription levels of *glaA* and *amyR*, with the gene-specific primers list below: for *glaA*, 5'-CACCGCAACGGACATTGTTT-3'(sense) and 5'-GGTGCCTGAGAATCACACCA-3'(antisense); for *amyR*, 5'-CGACGCCTTCTTCTTTCTTG -3'(sense) and 5'-CGGAGGAAACTGAGAGAACG -3'(antisense).

### 4: Determination of glucoamylase activity

0.5mL conidial suspensions of both original strain and mutants (1.2×10^6^ conidia /mL) were added into fermentation media for 12 h, 24 h and 48 h, respectively. The glucoamylase activity was assayed as described as following: a reaction mixture containing 250 μL of soluble starch dissolved by pH = 4.6 acetate buffer, and 20 μL of supernatant was incubated at 40°C for 30min. The released glucose was measured by SBA-40 biological sensor (China). One unit of glucoamylase activity was defined as the volume of enzyme which can form 1mg of glucose per hour under the incubated as above.

### 5: Analytical methods

Total sugar was assayed by Fehling reagent, and citric acid content of fermented samples by titration with 0.1429 M sodium hydroxide with 0.5% phenolphthalein as an indicator [20].

### 6: Statistical analysis

All exposure experiments were obtained from at least three times independently, and the data were recorded as the mean of the replications with standard deviation. The difference was expressed as *P < 0.05, **P < 0.01.

## Results

### 1: Effect of carbon ion irradiation on the colony appearance of A.niger

**Fig 1:**
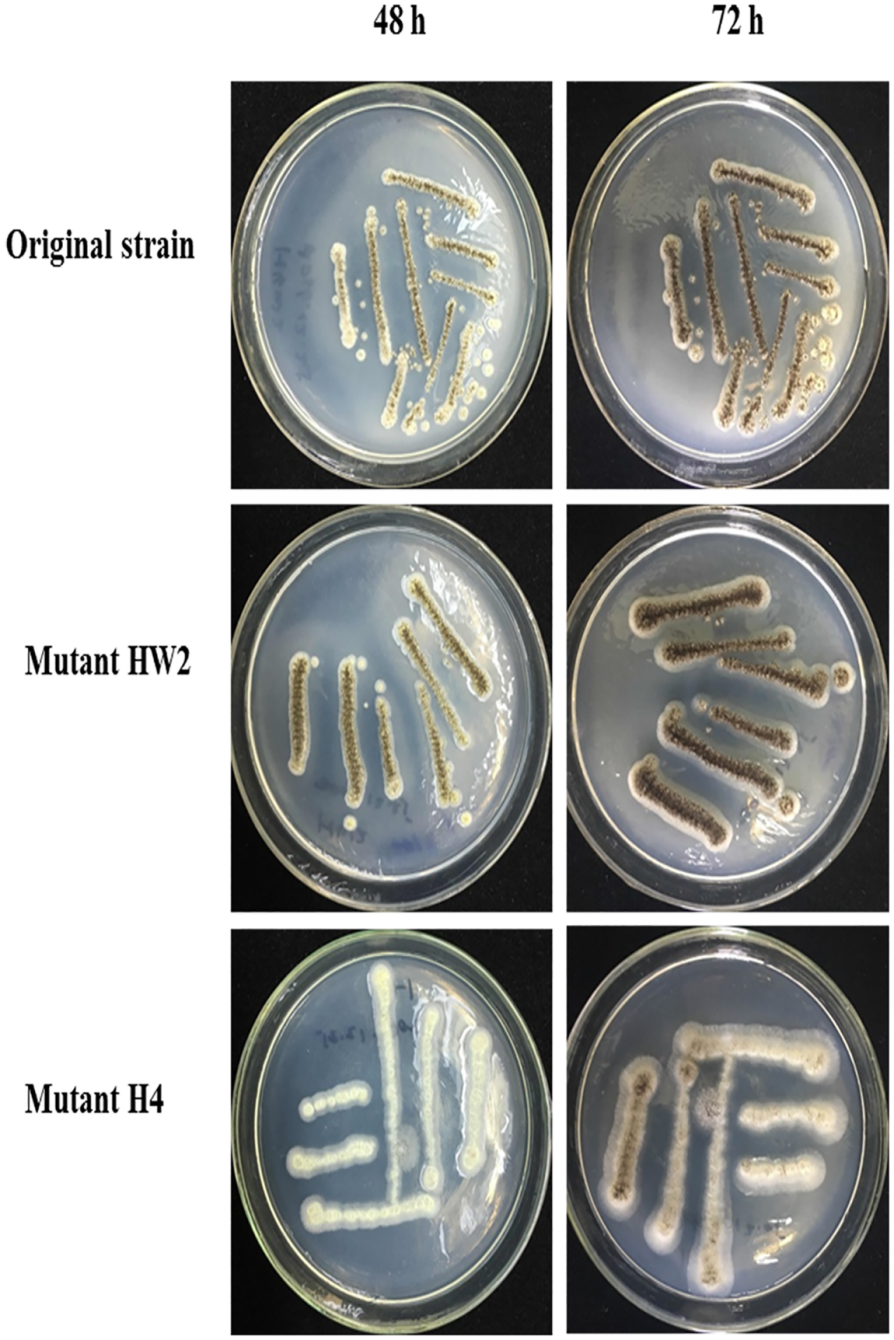
The colony appearance of *A*.*niger* after carbon ion irradiation.

The original strain was successively mutagenized with 80MeV/μ carbon ion beam (LET = 30keV/μ), and two different phenotypes *A*.*niger* mutants (HW2 strain and H4 strain) were obtained following screening protocol, confirmation in shake flasks and comparison with the original strain. After random mutagenesis treatment, colony appearance was one primary indicator to assess bacterial colony appearance [22]. Thus, to determine whether carbon ion beam could alter colony appearance, the *A*.*niger* spores of both original strain and mutants were cultivated on the solid starch medium for 48 h and 72 h. As shown in Fig 1, no clearly visible differences in morphology could be seen on PDA medium for mutant HW2 and mutant H4 compared with original strain, but especially a significant increase in spore formation rate was observed. Growth and, in particular, spore formation rate of mutant H4 was poor on PDA medium. No increase in spore formation rate was observed in mutant HW2.

### 2: Comparisons of the mutants and the original strain for citric acid production

**Fig 2:**
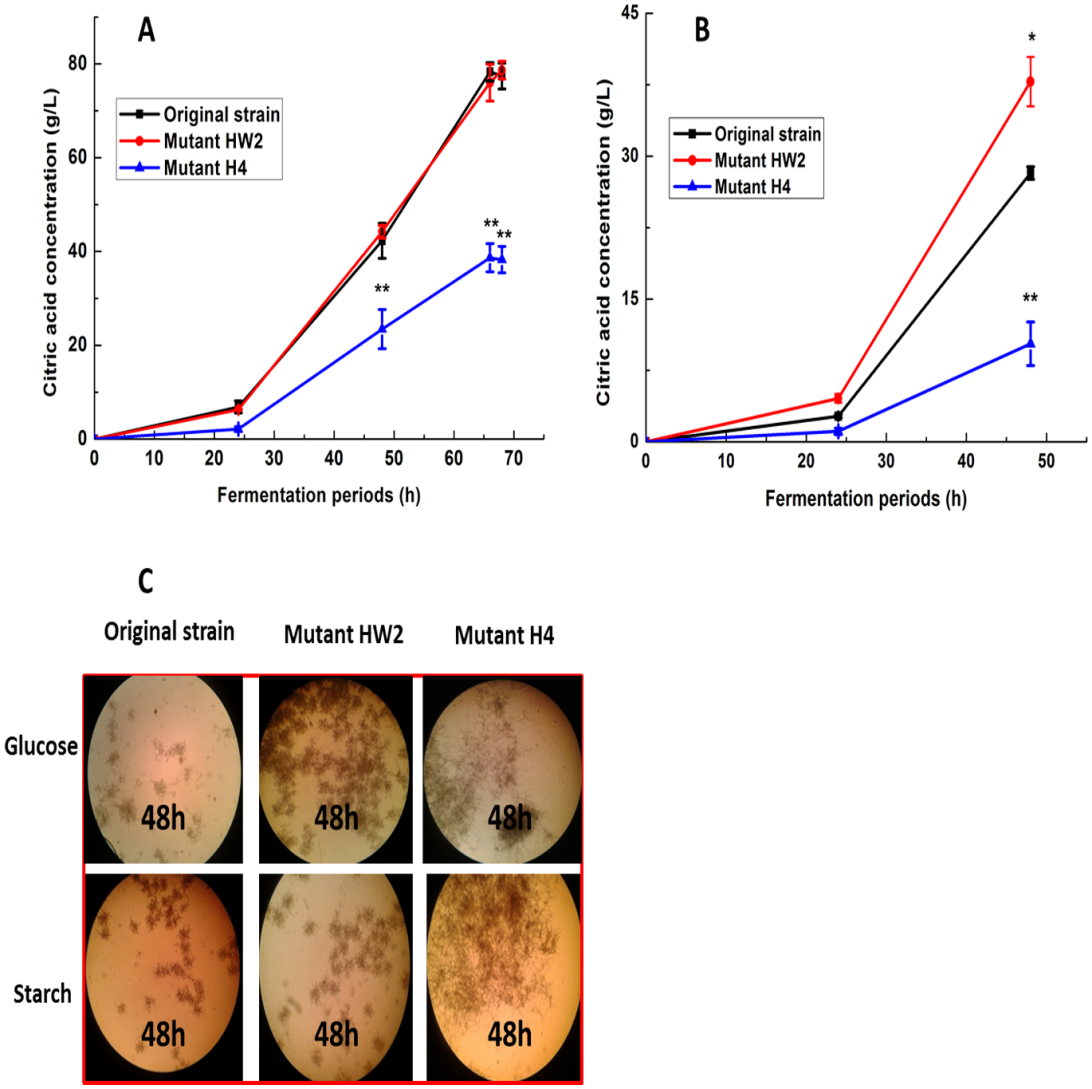
Effect of different carbon source on the citric acid accumulation of the original strain, mutant HW2, and mutant H4. Error bars indicate the standard deviation of the mean (n = 3), *P<0.05, **P<0.01, compared with the original strain.

Different carbon sources may result in variant morphological development and metabolic product accumulation. In order to investigate the effects of substrates on citric acid accumulation of original strain and mutants, various carbon sources (glucose and corn starch, respectively) were performed. As shown in Fig 2A, When grown in the media containing glucose as the sole carbon source supplement, HW2 mutant showed no obvious difference in citric acid accumulation compared with original strain, and H4 mutant also showed significantly subdued citric acid accumulation (P<0.01). When grown in the media containing corn starch as the sole carbon source supplement (Fig 2B), HW2 mutant showed significantly enhanced citric acid accumulation compared with original strain, which was obvious increase higher (P<0.05), whereas H4 mutant showed significantly subdued citric acid accumulation (P<0.01).

In addition, it was also observed that mutant H4 showed dispersed hypha morphology in fermentation broth, whereas both original strain and mutant HW2 showed pellets morphology when glucose or starch was the sole carbons source (Fig 2C). The results demonstrated that citric acid production in *A*.*niger* was significantly influenced by the morphology of the microcolonies during citric acid fermentation. Hence, we believed that high-LET carbon ion beam could cause alteration on the hypha morphology of *A*.*niger*, which may be an important factor resulting in different citric acid accumulation in *A*.*niger* mutants.

### 3: Effect of carbon ion irradiation on expression of glaA and amyR associated with starch hydrolysis of A.niger

**Fig 3:**
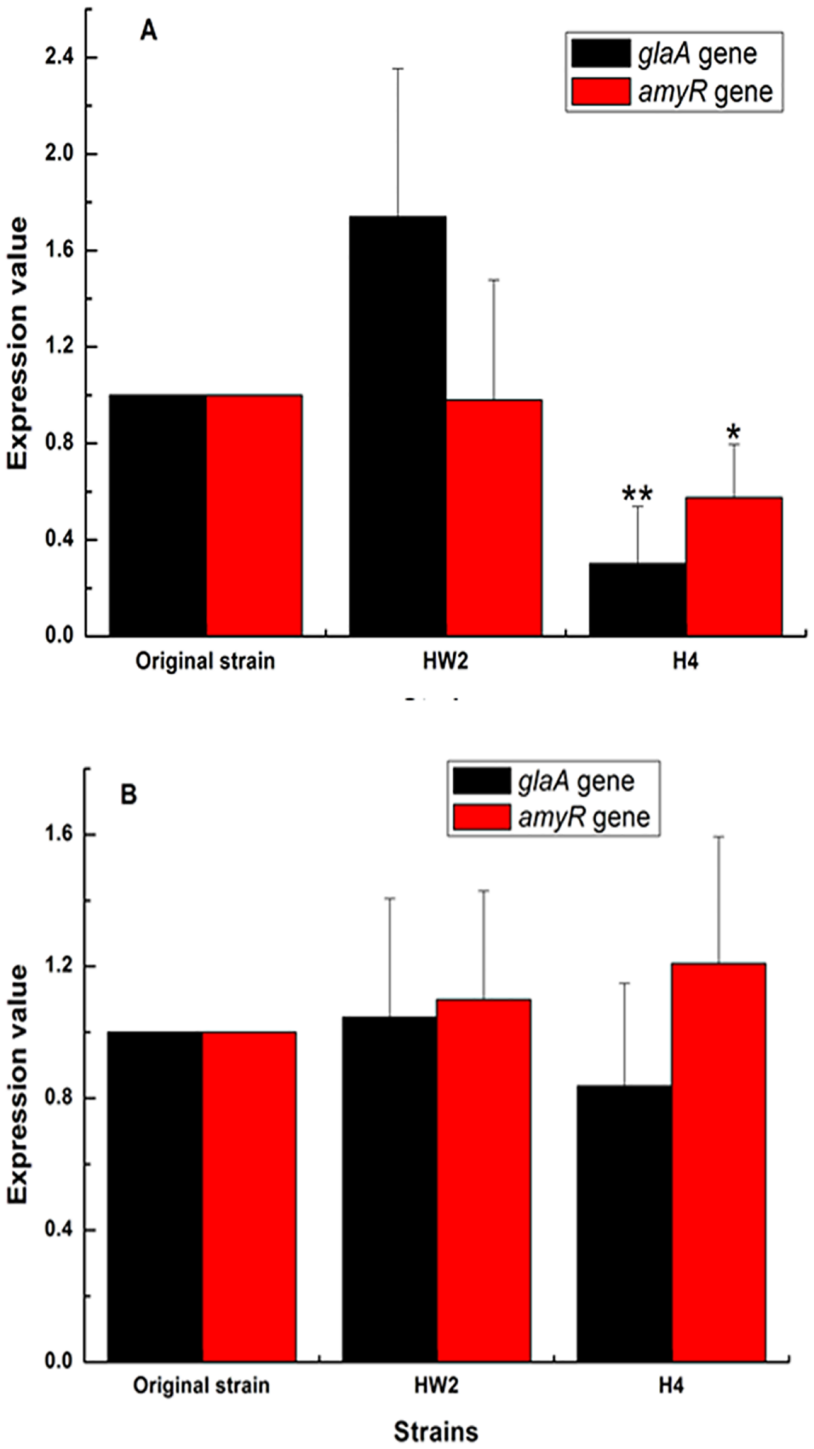
The real-time RT-PCR analysis of *glaA* and *amyR* transcript levels between the original strain, HW2, and H4 at different fermentation period: (A) 24h, (B) 48h. Values are the mean ±standard deviation of four independent biological replicate experiments (n = 4), *P<0.05, **P<0.01, compared with the original strain.

In present study, the expressions of *glaA* and *amyR* associated with starch hydrolysis of *A*.*niger* were detected by RT-PCR. The action gene (accession no. XM 001396558) was chosen as the housekeeping gene [23]. The results of Fig 3A showed that the transcript levels of both *amyR* and *glaA* in H4 strain were significantly lower than original strain. In the case of HW2 strain, the transcript level of *glaA* was higher than original strain, and the transcript level of *amyR* showed no expression difference at the early fermentation stage (24h). In the late fermentation period (48h), both HW2 and H4 strains showed no obvious difference in the transcript levels of both *glaA* and *amyR* expressions compared with original strain (Fig 3B). As we know, amylolytic system in *Aspergillus* can be induced by the presence of starch or maltose [24–27]. With the decrease of starch or maltose concentration, genes expression involving amylolytic system could be weaken. In present study, in the early fermentation period of 24h, with the existence of high starch or maltose concentration, HW2 strain showed higher *glaA* gene expression than original strain, indicating that HW2 strain can faster decompose starch into glucose than original strain. Meanwhile, H4 strain showed lower both *amyR* and *glaA* genes expressions than original strain, indicating that H4 strain can slower decompose starch into glucose than original strain. After 48h of fermentation, due to faster starch hydrolysis in earlier stage, lower starch or maltose could be existed in the fermentation broth of both HW2 strain and original strain than H4 strain, which would be the direct reason for no obvious difference in the transcript levels of both *glaA* and *amyR* expressions between original stain and mutants. From these results, we believed that H4 strain showed weakened starch hydrolysis ability, and HW2 strain showed enhanced starch hydrolysis ability compared with original strain.

### 4: Effect of carbon ion irradiation on glucoamylase activity

**Fig 4:**
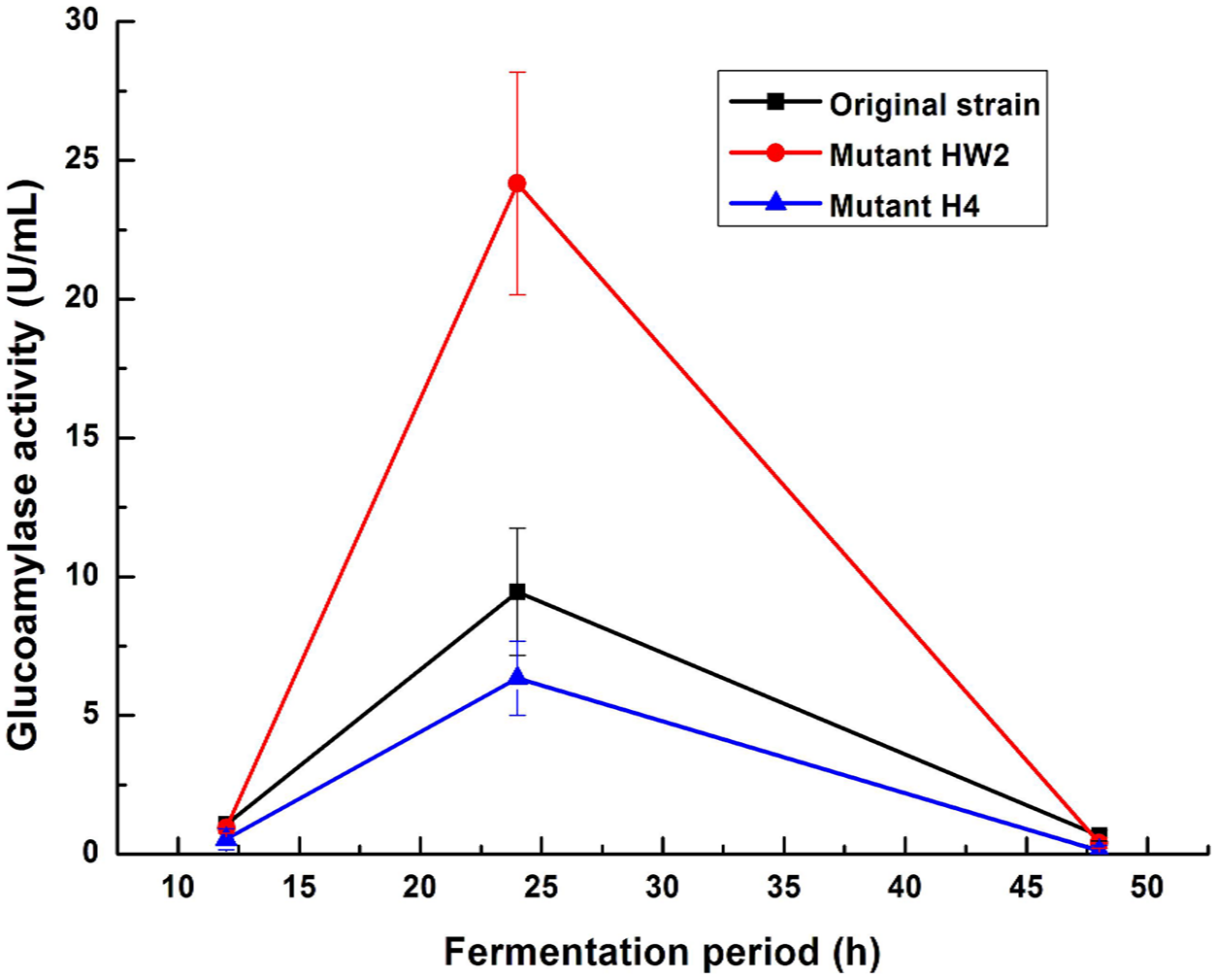
Determination of glucoamylase activity of original strain, mutant HW2 and mutant H4 in fermentation broth under different fermentation periods. Values are the mean ±standard deviation of four independent biological replicate experiments (n = 3).

We also detected relatively key enzyme activity associated with starch hydrolysis of *A*.*niger* to further test deduction described as above. Both glucoamylase (EC 3.2.1.3) and α-glucosidase (EC 3.2.1.20) can release glucose from maltooligosaccharides in starch hydrolysis process, but glucoamylase (EC 3.2.1.3) played a decisive role in saccharification during citric acid accumulation in *A*.*niger* [28]. In present study, after grown in fermentation media for 24h, glucoamylase activities of both original strain and mutants reached maximum (Fig 4). Glucoamylase activity of HW2 was higher than original strain whereas glucoamylase activity of H4 was lower. At the other fermentation periods (12h or 48h), both original strain and mutants showed very low glucoamylase activities, indicating that starch saccharification in *A*.*niger* also occurred in the early stage (24h). This result was in accordance with previous reported [8].

## Discussion

Heavy ion mutagenesis is a powerful and high-efficient tool that has been readily applied in mutagenesis of bacteria, fungi and microalgae [29]. Compared with low-LET (about 0.2-5KeV/μ) mutation induction techniques (X-rays or ɣ-rays)[30, 31], high-LET heavy ion beams can cause double-strand DNA break [32] owing to denser ionization along their trajectories, which induced different types of DNA damage, such as, translocations, inversions, insertion, and DNA deletions, which would be valuable for obtaining diverse phenotypic microbial mutants for functional genes research. For example, depending on three rice mutant with reduced Cd contamination induced by 320 MeV/μ carbon ions, Ishikawa, et al [33]identified OsNRAMP5 gene in rice responsible for reducing Cd uptake. Using RNA-Seq technology, Chen, et al [34] found the down-regulated and injured genes related to photosynthesis contributed to the growth-inhibited rice mutant induced by 40 Kev/μ N^+^ beam implantation. Through comparative analysis between wild type and utr319 rice mutant induced by carbon ions irradiation, TAKANO, et al [35]found that Os07g026510 gene in rice contributed to ultraviolet-B (UVB: 280–320 nm) tolerance. In the present study, heavy ion mutagenesis was conducted for the mutagenesis of filamentous ascomycete *A*.*niger* to obtained diverse phenotypic *A*.*niger* mutants with variable citric acid production capacity and investigations of the genetic mechanism in these mutants.

Firstly, comparison analysis of the mutants and the original strain for citric acid fermentation under different carbon sources showed that mutant HW2 exhibited more promising fermentation characteristic of producing citric acid than original strain using corn starch. Meanwhile, mutant H4 both showed subdued citric acid accumulation than original strain using corn starch or glucose. These *A*.*niger* mutants exhibited different citric acid accumulation using different carbon sources after heavy ion beam treatment, which was probably due to genetic mechanisms such as gene mutagenesis or transcriptional alteration involved in citric acid synthesis. For a long time, starch materials, such as corn starch and corn meal hydrolysate [8], have been widely used for productions of citric acid and other chemicals by fermentation in china due to its low price and stable supply. When starch materials were used as the substrate, two metabolic pathways were necessary for citric acid accumulation in *A*.*niger*. Firstly, starch materials cannot be used as the direct carbon source by *A*.*niger*, as it must be hydrolyzed into glucose through starch hydrolysis pathway before citric acid fermentation starts [8]. In this process, several glycoside hydrolase enzymes were contributing to degrading starch materials into fermentable sugars, such as α-amlyse, glucoamylase and α-glucosidase [36]. Secondly, citric acid biosynthesis involved glycolytic catabolism of glucose to pyruvate, of which one was converted to acetyl-CoA and the other one oxaloacetate and then finally condensing these two precursors to citric acid through TCA cycle [37]. Thus, it could be speculated that the contributions of mutagenesis breeding to the improvement of citric acid accumulation from starchy materials as a feedstock may were closed related with two metabolic pathways: starch hydrolysis pathway and glycolytic pathway. Therefore, it could be possible mechanism that mutant HW2 exhibited more promising fermentation characteristic of producing citric acid than original strain using corn starch possibly owing to its enhancement of the transcriptional expression levels involving starch hydrolysis pathway. Meanwhile, mutant H4 both showed subdued citric acid accumulation than original strain using corn starch or glucose possibly owing to its weakness of transcriptional expression levels involving starch hydrolysis pathway and glycolytic pathway.

In addition, *A*. *niger* HW2 formed bulbous hyphae with shorter hyphal branches than original strain, whereas *A*. *niger* H4 formed dispersed hypha morphology. These differences in morphology may influence the viscosity of fermentation broth and further affect hyphal respiration, as well as affected the production of metabolites and enzymes [38, 39]. The tight pellet form facilitated citric acid accumulation [40], and the dispersed hypha filamentous morphology reduced citric acid production and productivity.

**Fig 5:**
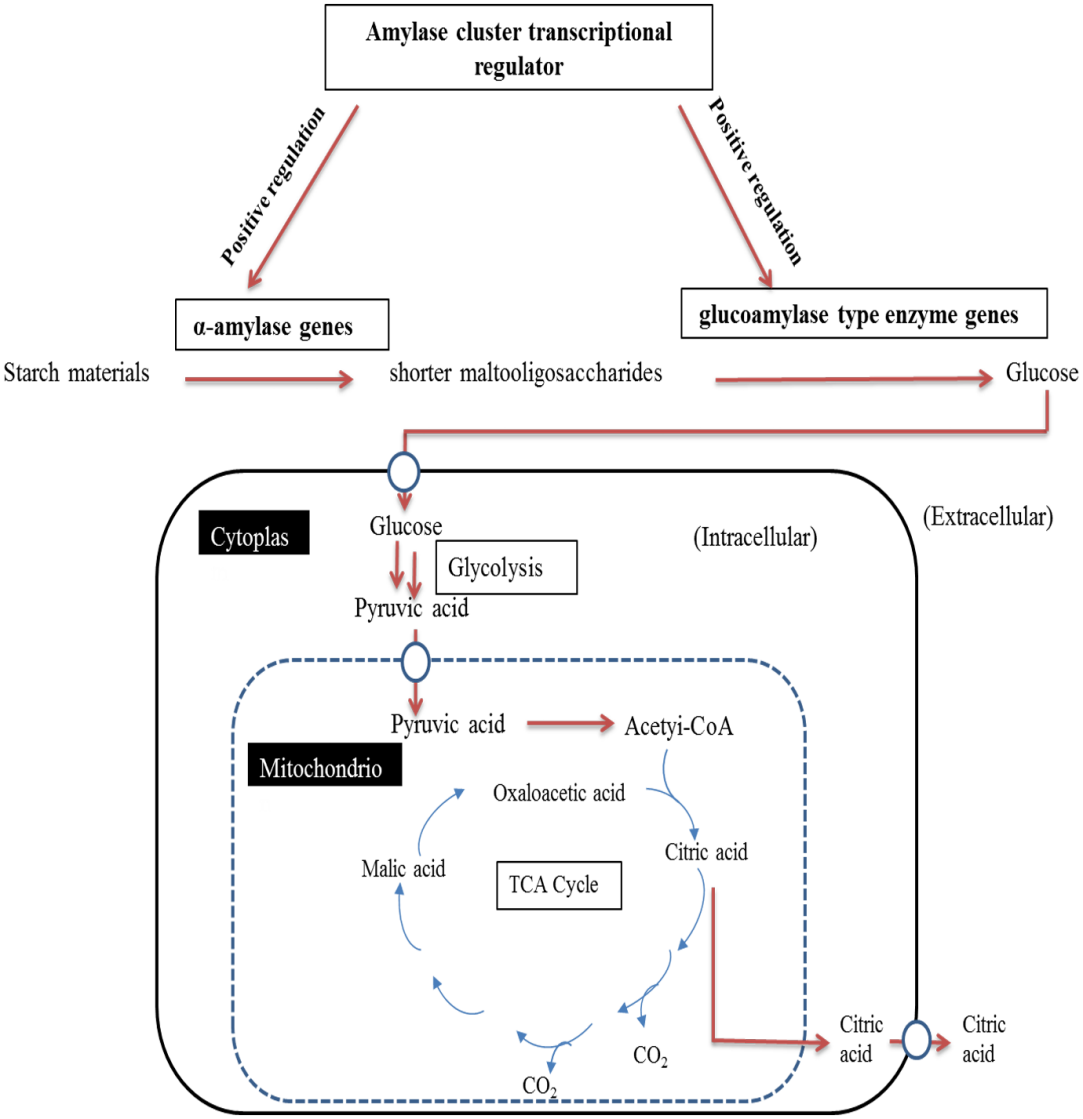
Schematic representation a simplified relationship between starch degradation and citric acid accumulation in *A*.*niger*.

Secondly, to explain the variation mechanism of citric acid production in the mutants, it was necessary to examine the associated genes transcription in starch hydrolysis pathway and glycolytic pathway. Though there were lots of studies on genes function involved in glycolytic pathway (such as *PFK*1, *PfkA* and *PkiA*) influencing citric acid accumulation in *A*.*niger*, detailed regulation mechanism by glycolytic genes was still not completely understood. Considering that the expression levels of genes involved in starch hydrolysis had a significant effect on citric acid accumulation in *A*.*niger* in previous study and starch hydrolysis pathway occurred before glycolytic pathway. Hence, we prior investigated whether high-LET carbon ions affected the expression levels of genes involved in starch hydrolysis in mutants in present study. *AmyR* encoded a pathway-specific activator essential for the starch hydrolysis in *Aspergillus*, which has been proved to bind to the CGGN_8_(C/A)GG sequence in various amylase promoters to activate relative genes transcription, such as α-and β-glucosidases, α-and β-galactosidases as well as genes encoding glucoamylases and α-amlyases [26, 41–43]. Overexpression or disruption of *amyR* in *A*.*niger* can enhance or weaken the utilization rate of starch [44]. *glaA* gene encoded protein involving in starch saccharification, and played an essential role in starch hydrolysis process, which can release glucose from non-reducing end of maltooligosaccharides [8, 36]. In addition, overexpression of *glaA* copies in recombinant *A*.*niger* strain, which can enhance glucoamylase activity compared with the original strain, has been studied in detail [8]. In present study, depending on RT-PCR experiment results, it was demonstrated that H4 strain showed weakened starch hydrolysis ability compared with original strain due to low transcript levels of both *amyR* and *glaA*, and HW2 strain showed enhanced starch hydrolysis ability compared with original strain due to high transcript levels of *glaA*. Fig 5 illustrated a simplified relationship between starch hydrolysis and citric acid accumulation [36, 44, 45], indicating that high starch hydrolysis ability can induce high citric acid accumulation in *A*.*niger*. This explained the results as shown in Fig 3, where the transcript levels of *glaA* in mutant HW2 were higher than original strain, and the transcript levels of both *amyR* and *glaA* in mutant H4 were significantly lower than original strain. This may help to reveal the mechanism of the high yield of citric acid production in mutants induced by carbon ion irradiation.

Finally, we also observed relative increase of glucoamylase activity in mutant HW2, and decrease of glucoamylase activity in mutant H4. Analysis of glucoamylase activity in our study demonstrated that high glucoamylase activity indicated fast citric acid accumulation ability in *A*.*niger*. Similar results were found by Suzuki et al [46] and Sarangbin, et al [7]. This may further revealed that different starch hydrolysis ability was the probable mechanism resulting in different citric acid accumulation in mutants induced by carbon ion irradiation.

In conclusion, the results in present study showed that high-LET carbon ion irradiation had a significant effect on colony appearance, citric acid accumulation and pellets morphology in *A*.*niger*, as well as significantly improved citric acid production by enhancing expression of gene associated closely with starch hydrolysis process, which were positive regulator for citric acid biosynthesis, and therefore open a new avenue for constructing genetically engineered *A*.*niger* mutants for high-yield citric acid accumulation in the future. More importantly, carbon ion irradiation presents an efficient alternative strategy to be developed to generate various phenotype microbe species mutants for functional genes research.
